# Antibiofilm Effects of Modifying Polyvinylidene Fluoride Membranes with Polyethylenimine, Poly(acrylic acid) and Graphene Oxide

**DOI:** 10.3390/polym16233418

**Published:** 2024-12-05

**Authors:** Mario Castillo-Ruiz, Constanza Negrete, Juan Pablo Espinoza, Iván Martínez, Leslie K. Daille, Christopher González, Bárbara Rodríguez

**Affiliations:** 1 Escuela de Tecnología Médica, Facultad de Ciencias de la Salud, Universidad Bernardo O’Higgins, General Gana 1702, Santiago 8370854, Chile; mario.castillo@ubo.cl; 2 Escuela de Química y Farmacia, Facultad de Medicina, Universidad Andres Bello, Sazié 2320, Santiago 8370134, Chile; 3 Facultad de Ciencias Naturales, Matemáticas y del Medioambiente, Universidad Tecnológica Metropolitana, Las Palmeras 3360, Ñuñoa 7800003, Chile; constanza.negretea@utem.cl; 4 CIBQA, Facultad de Ciencias de la Salud, Universidad Bernardo O’Higgins, Fábrica 1865, Santiago 8320000, Chile; jespinoza@ubo.cl; 5 Departamento de Ciencias Químicas y Biológicas, Facultad de Ciencias de la Salud, Universidad Bernardo O’Higgins, General Gana 1702, Santiago 8370854, Chile; ivan.martinez@ubo.cl; 6 Centro GEMA-Genómica, Ecología & Medio Ambiente, Universidad Mayor, Camino La Pirámide 5750, Santiago 8580745, Chile; lkdaille@gmail.com; 7 CIRENYS, Escuela de Química y Farmacia, Facultad de Ciencias Médicas, Universidad Bernardo O’Higgins, General Gana 1702, Santiago 8370854, Chile; gonzalezponcechristopher@gmail.com

**Keywords:** membranes, filtration, polyelectrolytes, antibacterial, antibiofilm, LBL assembly

## Abstract

Biofouling in membrane filtration systems poses significant operational challenges, leading to decreased permeate flux. The aim of this work was to study the anti-biofilm properties of new nanofiltration membranes produced via layer-by-layer, LBL, assembly by coating a polyvinylidene fluoride (PVDF) support with a polyethylenimine (PEI) and poly(acrylic acid)/graphene oxide (PAA-GO) mixture. The membranes were characterized according to contact angle, scanning electron microscopy (SEM), atomic force microscopy and their Z-potential. Biofilm quantification and characterization were carried out using crystal violet staining and SEM, while bacterial viability was assessed by using colony-forming units. The membrane with three bilayers ((PAA-PEI)_3_/PVDF) showed a roughness of 77.78 nm. The incorporation of GO ((GO/PAA-PEI)_3_/PVDF) produced a membrane with a smoother surface (roughness of 26.92 nm) and showed salt rejections of 16% and 68% for NaCl and Na_2_SO_4_, respectively. A significant reduction, ranging from 82.37 to 77.30%, in biofilm formation produced by *S. aureus* and *E. coli* were observed on modified membranes. Additionally, the bacterial viability on the modified membranes was markedly reduced (67.42–99.98%). Our results show that the modified membranes exhibited both antibiofilm and antimicrobial capacities, suggesting that these properties mainly depend on the properties of the modifying agents, as the initial adherence on the membrane surface was not totally suppressed, but the proliferation and formation of EPSs were prevented.

## 1. Introduction

Water scarcity has increased worldwide over the past year and, considering that only 1% of Earth’s total water supply is freshwater [1], the use of alternative water sources has become essential. These sources include wastewater, seawater and brackish water [2]. Among the processes used to effectively remove salts from seawater and brackish water, pressure-driven membrane separation stands out, with reverse osmosis (RO) considered the most efficient industrial desalination method [3,4]. However, RO requires high operating pressures (900–1000 psi), leading to significant operational costs. In this context, low-pressure processes like nanofiltration (NF) are regarded as promising alternatives for brackish water desalination.

During membrane filtration, different types of fouling can occur on membrane surfaces, including organic fouling, scaling fouling, and biofouling. Biofouling, which arises from bacterial adhesion and the subsequent deposition of extracellular materials that form a biofilm, is a primary factor impacting membrane lifespan [5]. Biofouling is caused by bacteria attached to the membrane surface and the subsequent deposition of extracellular material that produces a biofilm. Biofilms are stable ecosystems of bacterial aggregates encased in an extracellular matrix [6], comprising bacterial cells embedded in a heterogeneous mix of extracellular polymeric substances (EPSs), structural proteins, polysaccharides and extracellular DNA [7]. Biofilm formation occurs in various natural, clinical, and industrial environments, where bacteria adhere to both biotic and abiotic surfaces [8]. The bacteria in biofilms show an increased tolerance to external stresses [9], making biofilms challenging across sectors such as food processing, industrial manufacturing, marine industries, sanitation and water distribution [10].

Controlling biofilms on NF membranes is challenging. Strategies to prevent bacterial adhesion include mechanical removal [11], chemical treatment [12] or designing surfaces to prevent adhesion [13,14]. Mechanical removal is used once a biofilm is established, but this implies a delay in operation time and possible damage to the membrane surface. Chemical treatment uses antimicrobial agents as chlorine that produce structural damage on the polymer membrane’s surface [12]. To reduce biofouling, surface modification approaches, such as the integration of biocidal nanomaterials during material fabrication [15,16,17,18,19] or the application of hydrophilic polymers through coating, grafting or layer-by-layer (LBL) self-assembly [20,21], have been explored.

The LBL self-assembly of polyelectrolyte multilayers (PEMs) is a promising technique for creating NF membranes, offering advantages such as simplicity, versatility, and nanoscale control [22,23]. The use of polyethyleneimine (PEI) and polyacrylic acid (PAA) has shown to effectively modify polyacrylonitrile (PAN) membrane surfaces via LBL assembly, achieving a moderate-to-good rejection of mono- and divalent salts [24]. Polyvinylidene fluoride (PVDF), a widely used polymer in the membrane industry, has excellent mechanical and thermal stability [25,26,27]. While PVDF is commonly applied in ultrafiltration and microfiltration, its ion removal capacity is limited. However, PVDF membranes modified with polyelectrolytes using the LBL assembly technique have emerged as an effective approach for enhancing salt rejection in water purification applications by increasing electrostatic interactions and ion selectivity [28,29,30]. Accordingly, PVDF has gained interest as a support for NF membranes fabricated through PEM self-assembly. Positively charged NF membranes created by coating PVDF with PEI have demonstrated a good salt rejection rate [31], while PVDF membranes coated with polyvinyl alcohol (PVA) and silver nanoparticles have shown a high water flux, moderate salt rejection and antibacterial properties [32]. However, studies of the antibiofilm properties of these membranes are still limited. On the other hand, graphene oxide (GO) is a relevant material that has been used as an additive in PEM solutions for membrane filtration modified using the LBL method. The incorporation of GO as an interlayer to coat the PAN surface with PEI produced NF membranes with high divalent ion rejection rates [33]. In another approach, the successive coating of a PAN substrate with a mixture of PEI-GO and only GO solutions produced NF membranes with high MgCl_2_ selectivity removal rates [34]. Whilst the incorporation of silane-GO into PAA has been used to produce modified polyvinyl chloride (PVC) membranes by LBL coating with chitosan (CS) as a positive PEM and PAA as a negative PEM [35]. These membranes were selective for monovalent ion separation. In addition, GO has attracted significant attention for its antimicrobial properties and potential as a biofilm inhibitor [36,37,38]. The unique structure of GO, characterized by its high surface area and functional groups, allows for effective interactions with microbial membranes, leading to increased membrane permeability and cell death [39]. Studies have demonstrated that GO exhibits antibacterial activity against a wide range of pathogens, including both Gram-positive and Gram-negative bacteria [40]. Its mechanisms of action include oxidative stress induction and the disruption of cellular functions, making GO a versatile agent in combating microbial contamination [41,42]. Furthermore, this material improves the mechanical strength, permeability and selectivity of membranes, making them more effective in water treatment applications [43,44]. Studies have shown that the addition of GO or rGO (reduced graphene oxide) can significantly reduce membrane fouling, thus prolonging operational lifespans and reducing maintenance costs [45]. The unique properties of graphitic materials, including their high surface area and tunable chemical functionalities, facilitate enhanced interactions with water molecules and solutes, leading to improved separation [46,47,48,49]. Furthermore, the integration of these materials into polymeric membranes has been found to increase their hydrophilicity, which contributes to better antifouling characteristics [50,51]. In fact, different GO structures have been incorporated into PVDF-UF membranes via phase inversion methods to promote their antibacterial properties [38]. In this regard, the research reported on PVDF membranes modified with polyelectrolyte multilayers (PEMs) or graphene oxide (GO) has addressed the study of the bactericidal properties of biofouling materials [16,38,52,53,54,55]; no precedent exists in the literature for combining polyelectrolytes like PEI and PAA with GO to develop NF membranes with antibiofilm capabilities. The aim of this work was to study the anti-biofilm properties of new NF membranes produced via the LBL assembly technique, combining PEI with a PAA-GO mixture on UF-PVDF supports. Taking advantage of the antimicrobial properties, functional groups, and surface charges of the polyelectrolytes selected. This novel approach seeks to advance the development of membranes with enhanced antibiofilm performances.

## 2. Materials and Methods

Materials and Chemicals. Polyvinylidene fluoride (PVDF, Snyder 1812 BN UF) membranes with a molecular weight cutoff of 50,000 Da were used as a substrate, and were provided by the STERLITECH corporation (Auburn, WA, USA). Branched polyethylenimine (PEI) with an average Mw of ~25,000, Poly(acrylic acid) (PAA) with an average Mv of ~450,000 and graphene oxide powder (15–20 sheets, 4–10% edge oxidized) were purchased from Sigma Aldrich (Darmstadt, Germany); ACS-grade sodium sulfate (Na_2_SO_4_) was bought from Merck (Darmstadt, Germany), sodium chloride (NaCl) and isopropyl alcohol (CH_3_CH(OH)CH_3_) were bought from Winkler (Región Metropolitana, Chile), Glutardialdehyde (C_5_H_8_O_2_) (GA) solution 25% *w*/*w* was bought from Scharlau (Barcelona, Spain) and demineralized water (DI) with a conductivity less than 2 μsiemens/cm was purchased from Aguas de la Fuente (Región Metropolitana, Chile).

Membrane modification by LbL Assembly. The modification of the PVDF support was carried out following the methodologies of Y. Liu et al. [24] and C. Wang et al. [56] with some modifications. The PVDF substrate was carefully cut to the dimensions of 6.5 × 6.5 cm. Before coating the LbL assembly, the PVDF substrate was washed following the methodology described by Wang et al. For this purpose, the PVDF substrate was immersed in 25 % *v*/*v* isopropyl alcohol for 30 min to eliminate any possible protective coating or preservatives. The cleaned membrane was then immersed in demineralized water twice, each time for 60 min, to remove residual isopropyl alcohol and stored in demineralized water overnight at 4 °C [24,56]. Polycation and polyanion coating solutions were prepared by dissolving 1 g/L PEI and 0.2 g/L PAA in a 0.1 M NaCl solution, respectively, and stirring continuously. And, a 5 g/L GO stock solution was dispersed in a sonicator bath for 15 min at room temperature to prepare a 0.19 g/L GO-PAA solution.

The prepared polyelectrolyte coating solutions were applied to the PVDF substrate by dipping with the help of a dip coater, programmed with a dipping time of 10 min and drying time of 2 min. First, the cleaned PVDF substrate was placed in a square glass frame and, on top of this, a Teflon square frame was firmly clamped with only the surface of the active layer exposed; 100 mL of the prepared PEI solution was added to a glass container and coatings were carried out with the dip coater using the conditions mentioned above. After ten minutes, the membrane was rinsed with a 0.1 M NaCl solution (i.e., an electrolyte with an identical concentration to that used for polyelectrolytes) for two minutes to remove any excess polyelectrolyte. After rinsing, a second layer was attached through immersion contact (with the same conditions mentioned above) between the PAA anionic polyelectrolyte solution and the previous one for 10 min to induce an electrostatic attraction between the macromolecules of the polycation and the polyanion. Subsequently, excess PA was removed as described above. Thus, the first PEI-PAA bilayer was formed on the PVDF substrate. This step must be repeated according to the number of bilayers. After the appropriate number of bilayers had been formed, the membranes were cured in an oven at 65 °C for 5 min and cross-linked with a 3% *v*/*v* GA solution for 20 min. Finally, the membranes were washed with demineralized water for 3 min. The modified membranes were identified with code (PAA-PEI)n/PVDF, where n corresponds to the number of bilayers (n = 1 and 3).

GO incorporation was carried out according to the methodology previously reported [56] with some modification. For the membranes modified with GO, the previous procedure was repeated; first, the PVDF membrane was modified through immersion with a dip coater in a 1 g/L PEI solution and the membrane was washed with a NaCl solution and then immersed in a (GO/PAA) solution that had been previously sonicated (with GO concentration of 0.19 g/L) before being rinsed with NaCl solution. Thus, a GO/PAA bilayer on a PVDF substrate was formed. This step was repeated 3 times to obtain 3 bilayers (GO/PAA)_3_. And, the curing, cross-linking and washing procedure mentioned above was repeated. Therefore, the GO-modified membrane was identified as (GO/PAA-PEI)_3_/PVDF.

Membrane Characterization. Scanning electron microscopy (SEM) was used to characterize the surface morphology of the prepared multilayer membranes. Prior to the SEM analysis, the samples were coated with gold to improve their conductivity, using a Cressington 108 (TedPella, Redding, CA, USA) sputter coater. The samples were observed under a high-resolution Inspect-F50 scanning electron microscope (FEI, Eindhoven, The Netherlands) with magnifications of 25,000X at 5.00 kV. Three randomly selected areas (upper, central and lower) on each membrane were analyzed to obtain representative images. The water contact angle on the membrane surfaces was measured by using the sessile drop method. Three cuts of each type of membrane were taken and glued to a slide with double-stick tape. On each piece of membrane, 3 to 5 microdrops (5 µL) were deposited in different areas. Images were obtained using a digital microscope sg105423 (Tecnolab, Shanghai, China), a 500× zoom lens, and a resolution of 8 µm/pixe. These images were processed in the ImageJ software 2.9.0, measuring the angle in the two zones of the drop (zone A and zone B, see Supplementary Materials Figure S4). Fourier transform infrared (FTIR) spectroscopy (Jasco FTIR-4X, Tokyo, Japan) equipped with a single-reflection attenuated total reflectance (ATR) was used to analyze the chemical composition bands of the PVDF membranes and to confirm the presence of surface organic groups on the membranes modified layer by layer. Membrane surface roughness was assessed through atomic force microscopy (AFM) (Bruker Innova, Karlsruhe, Germany) using mode tapping. Four different zones with areas of 2.5 μm × 2.5 μm were studied for each sample and one-way ANOVA was applied for data treatment (average value, roughness and RMS). The zeta potential of the membrane surface was evaluated using the streaming potential method with the Electrokinetic Analyzer for Solid Surfaces (SurPASS 3-Anton Paar, Graz, Austria). NaOH and HCl 0.1 M solutions were used for pH adjustment and KCl 0.001 M was used as an electrolyte solution.

Membrane Performance Measurements. The performance of the membranes was evaluated by measuring water flux and salt retention, according to previous reports [57,58,59] with some modifications. All measurements applying hydraulic pressure were performed using a crossflow configuration with an effective membrane area of 20.6 cm^2^.

Water flux. The water flux of membranes was measured with distilled water at an applied transmembrane pressure of 100 psi (6.9 bar) using the setup described above. The membrane was compacted for 30 min, and then the permeate volume was collected; a stopwatch was used to measure the time. Using the time and volume of permeate, the water flux can be calculated as shown in Equation (1):

(1) $$V=\frac{J}{A\cdot ∆t\cdot P}$$

where *J* (L/m^2^.h.ba) is the membrane flux, *V* (L) is the volume of permeate water, *A* (m^2^) is the membrane area and Δ*t* is the permeation time and *P* (bar) is work pressure. The experiments were carried out at room temperature.

Salt Retention. The retention of NaCl and Na_2_SO_4_ was determined at a concentration of 10 mM in distilled water. These measurements were performed on the same setup as described above, under crossflow conditions at an applied pressure of 100 psi (6.9 bar). Samples were collected after 60 min, measuring volume and conductivity of the permeate and feed solution as a function of time.

Retention (%R) was determined using the relationship presented in Equation (2):

(2) $$\%R=\frac{∆C}{C_{feed}}\cdot \;100$$

where Δ*C* and *C* feed correspond to the difference between the feed and permeate conductivity and the feed conductivity, respectively.

Bacterial strain, media and growth conditions. The *Staphylococcus aureus* strain was isolated on blood agar. The *Escherichia coli* strain was isolated on MacConkey agar at 37 °C for 18–24 h. Bacteria were grown in nutritive broth at 37 °C with agitation for 18-24 h. SCDLP broth (casein peptone 17.0 g/L, soybean peptone 3.0 g/L, sodium chloride 5.0 g/L, disodium hydrogen phosphate 2.5 g/L, glucose 2.5 g/L and lecithin 1.0 g/L) was used for the evaluation of antimicrobial activity. Plate count agar (yeast extract 2.5 g/L, tryptone 5.0 g/L, glucose 1.0 g/L and agar powder 15.0 g/L) was used to determine the quantity of viable bacteria. Nutrient broth (peptone 5 g/L, beef extract 3 g/L, sodium chloride 5 g/L) was used for biofilm development.

Biofilm formation assay. Biofilms were produced in pre-sterilized, 24-well flat-bottomed polystyrene microtiter plates. For biofilm development, an approximately 15 mm diameter UV-sterilized membrane was deposited in each well, containing 1.9 mL of nutrient broth. Then, 100 μL of a standard cell suspension (McFarland 0.5) was added and incubated at 37 °C without agitation for 48 h. As a control, a medium without bacteria was used. After incubation, the culture supernatant was removed, and wells were washed three times with distilled water. Then, the membranes were used for SEM, Confocal visualization or quantification using crystal violet. The membranes used for microscopy visualization were immersed in 2.5% glutaraldehyde buffer for 3 h at 4 °C, dried under vacuum at 40 °C for 24 h and then examined using SEM. For the biofilm quantification, the biofilm was stained with 1 mL of a 0.1% crystal violet solution for 15 min and washed three times with distilled water. To solubilize the crystal violet, 1 mL of 95% ethanol was added per well and incubated for 20 min at room temperature. The crystal violet solubilized in ethanol was transferred to a new multi-well plate, and its absorbance was measured at 595 nm using a Microplate Reader (Tecan Infinite 200 PRO, Zurich, Switzerland). OD data were first normalized to the mass of each corresponding membrane segment to ensure accurate comparisons were made. To correct for the intrinsic retention of crystal violet, the OD values obtained from non-inoculated controls were subtracted from those of the inoculated samples. Subsequently, the data were relativized to the PVDF membrane, considering it as a 100% biofilm quantification.

Confocal microscopy. To observe the bacterial viability within the biofilm, the membranes were prepared as mentioned above. After 48 h incubation, the membranes were taken and washed three times with PBS 1X and then stained by immersion in a 5 mL of deionized water with 5 μL of 6 M Syto9 (excitation 485 nm and emission 530 nm) and 5 μL 30 M propidium iodide (excitation 485 nm and emission 630 nm) (LIVE/DEAD BacLight™ Bacterial Viability L13152, Molecular Probes, Invitrogen). Samples were washed again three times in PBS 1X and were studied using a confocal microscope (FluoView™ FV1000, Confocal laser scanning microscope, Olympus Corporation, Tokyo, Japan) using the 60X objective. Image analysis was performed using the Fiji software 2.9.0. All images were processed uniformly. First, they were converted to 8 bit grayscale. Then, the background was removed using the “Subtract Background” tool with a rolling ball radius of 7 pixels. The images were then converted to binary, and an automated particle count was conducted using the “Analyze Particles” tool. Cell classification was carried out as follows: all particles present in the green channel were classified as live cells, while particles in the red channel were counted as dead cells. The percentage of live cells was calculated considering the number of green particles relative to the total number of green and red particles.

Evaluation of antimicrobial activity. An inoculum of *Staphylococcus aureus* or *Escherichia coli* was prepared at a bacterial concentration ranging from 2.5 × 10^5^ CFU/mL to 10 × 10^5^ CFU/mL in 1/500 diluted nutrient broth. Then, 100 µL of each inoculum was applied onto 3.25 × 3.25 cm sections of the PVDF control membrane, (PAA-PEI)_3_/PVDF or (GO/PAA-PEI)_3_/PVDF, which were subsequently covered with a 2.25 × 2.25 cm polypropylene film. These samples were then incubated for 24 h at 37 °C.

Following incubation, bacteria were recovered with 10 mL of SCDLP broth, which was used for quantification through serial dilutions. Dilutions were plated and cultured on agar count plates for 48 h to determine the quantity of viable bacteria. To calculate the initial quantity of the viable bacteria, bacteria from two control membranes were recovered with SCDLP broth immediately after inoculation.

Statistical analysis. Statistical analyses were conducted using GraphPad Prism 9.0.0 software. For biofilm assays, the Kruskal–Wallis test was applied. For the percentage of live cells, a one-way ANOVA was applied followed by Tukey’s multiple comparisons test.

## 3. Results and Discussion

### 3.1. Membrane Characterization

#### 3.1.1. Hydrophilicity, Morphology and Roughness of Membrane Surface

The control and prevention of biofouling on the surface of RO/NF membranes is a complex issue and it can be addressed in different ways. One of them is through membrane surface modification. These strategies include an anti-adhesion approach, anti-microbial approach and the incorporation of materials that have a combination of the aforementioned properties. Regarding the anti-adhesion approach, it has been reported that the formation of biofouling is influenced by the hydrophilicity, roughness and charge of the membrane surface. Thus, the synergistic effect of the aforementioned surface properties could promote biofouling control through the production of membranes modified to have smoother surfaces, more hydrophilic surfaces and surfaces with a charge similar to the microorganism’s cell membrane [60,61].

**Figure 1:**
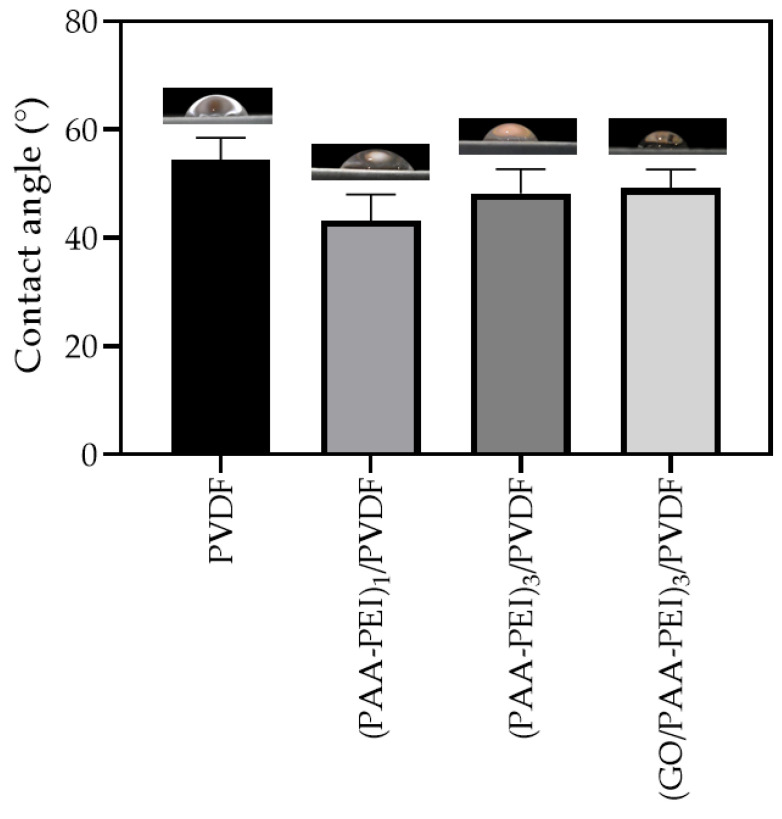
Contact angle of membranes. A one-way ANOVA was performed on the results. The membranes showed contact angles with significant differences, except for both membranes with three bilayers.

In order to evaluate the hydrophilicity of the membranes used in this study, water contact angle measurements were carried out. As seen in Figure 1, the PVDF membranes showed a moderate hydrophilicity with a contact angle of (54 ± 4)° due to the presence of the polar β-phase which was confirmed by ATR (Supplementary Figure S1). The polar β-phase induces the hydrophilicity of PVDF [62]. It is known that most PVDF membrane surfaces are characterized as being hydrophobic. However, it has been reported that the increase in the β-phase in the PVDF structure promotes an increase in hydrophilicity due to the crystal structure in which the C-F polar bonds with a high dipole moment are oriented in the same direction, producing an increase in the dipole moment of the polymer [62]. The deposition of a bilayer of PAA-PEI on the PVDF substrate produced membranes that were more hydrophilic than the unmodified membrane, with decreasing contact angles up to (43 ± 5)°. This behavior could be attributed to the new functional groups on the membrane’s surface produced by a reaction between the hydroxyl groups present in PAA with amine groups of PEI through an electrostatic interaction or the formation of H-bonds [63]. Subsequently, the deposition of three bilayers led to an increase in the contact angle of 11.6%, and the (PAA-PEI)_3_/PVDF membrane showed a contact angle of (48 ± 4)°. The addition of GO to the PAA coating solution produced the (GO/PAA-PEI)_3_/PVDF membrane with a hydrophilicity similar to the membranes modified with three bilayers without GO. In this regard, it is possible to see that the nature of the GO sheets does not influence the hydrophilicity. Finally, the modification of the PVDF membrane carried out in this study produced membranes that were more hydrophilic than the pristine membrane. Several works have reported the use of hydrophilic polymer to modify membrane surface in order to confer anti-biofouling properties [64]. The presence of polar functional groups on the membrane’s surface due to the hydrophilic polymer increases the interaction with the water and decreases the interaction with foulants including microorganisms [65,66].

**Figure 2:**
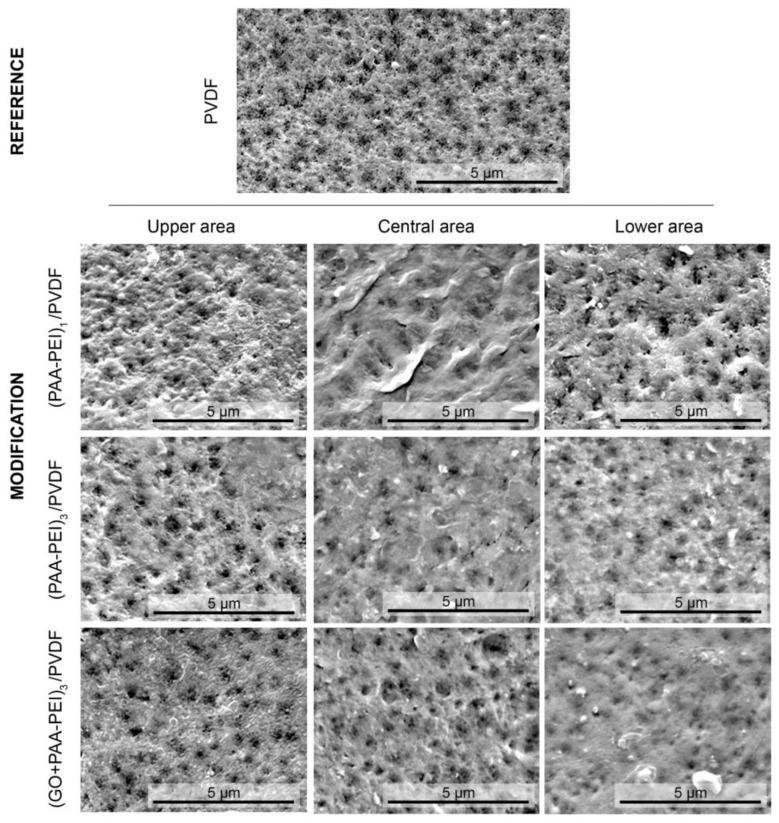
Scanning microscope (SEM) images of the PVDF membrane and the modified PVDF multilayer membranes. The polyvinyl fluoride membrane was used as reference. The following modifications were observed: PVDF membrane modified with three bilayers of PEI and PAA ((PAA-PEI)_3_/PVDF) and PVDF membrane modified with three bilayers of PEI and GO/PAA ((GO/PAA-PEI)_3_/PVDF). All images were taken with a magnification power of 25,000×.

**Figure 3:**
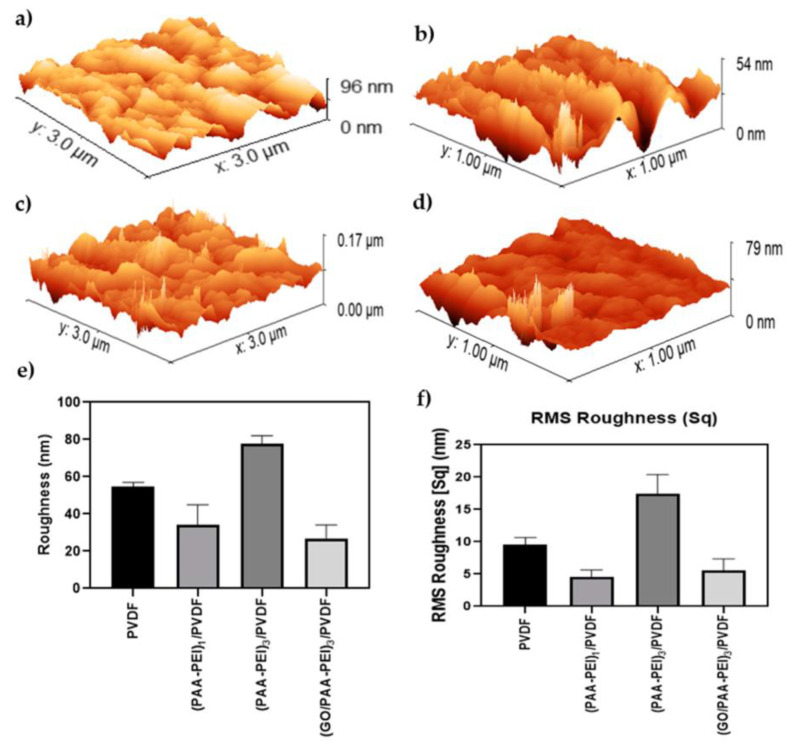
AFM images of membranes. (**a**) PVDF; (**b**) (PAA-PEI)/PVDF; (**c**) (PAA-PEI)_3_/PVDF; (**d**) (GO/PAA-PEI)/PVDF; (**e**) average roughness values of different membranes; (**f**) RMS of different membranes. A one-way ANOVA was performed on the results, showing that all membranes have average roughness values that show significant differences with respect to the pristine membrane.

SEM and AFM images of the membrane surfaces were taken in order to understand the morphology and roughness of the synthesized membranes. Figure 2 shows the SEM images obtained from three different areas (upper, middle and lower) on each membrane. It is possible to observe that the pristine PVDF membrane has a porous and homogeneous morphology. The coating of the membrane surface with one and three bilayers of PAA-PEI led to a decrease in the porosity. The images of the three different areas of membrane surfaces showed a very similar morphology between the central and lower areas, with slight differences with respect to the upper area. Also, the formation of some channels composed of smaller pores is observed. Finally, the modified membrane incorporating GO into the PAA coating solution ((GO/PAA-PEI)_3_/PVDF)) (Figure 2) showed a honeycomb-like morphology, with a heterogeneous porosity compared to the pristine PVDF membrane. The AFM images (Figure 3) show that the PVDF membrane has a roughness of (54.65 ± 2.35) nm. Coating with one PAA-PEI bilayer produced a membrane with a roughness of (34.31 ± 10.68) nm which is 1.6 times lower than the roughness of the pristine membrane. The increase in the number of bilayer to three ((PAA-PEI)_3_/PVDF) produced an increase in roughness to up to (77.78 ± 4.24) nm. The addition of graphene oxide to the PAA during the modification of the membrane with three bilayers produced a membrane with a smoother surface (roughness of (26.92 ± 7.19) nm), which may suggest an ordered arrangement of the GO sheets on the surface. A statistical study of membrane surface roughness was carried out. Thus, the modified membranes showed significantly different roughness values (*p* < 0.02) to the pristine membrane. When performing multiple comparisons, only the membranes with one and three bilayers with GO showed roughness values without significant differences. Regarding RMS, the membrane modified with three bilayers and GO showed a significantly lower roughness value than the same membrane without GO (*p* < 0.0001). Likewise, an increase in the number of bilayers without GO significantly increased the roughness (*p* < 0.0001). The roughness of the membranes with one bilayer and three bilayers with GO did not show significant differences (*p* < 0.9166) between them or with respect to the pristine membrane (*p* < 0.1634). Only the modification using three bilayers without GO showed a significantly higher value than the unmodified PVDF. Membranes with smoother surfaces are desirable to avoid the adhesion of bacteria and the subsequent formation of biofilms [67].

#### 3.1.2. Membranes’ Performances

**Figure 4:**
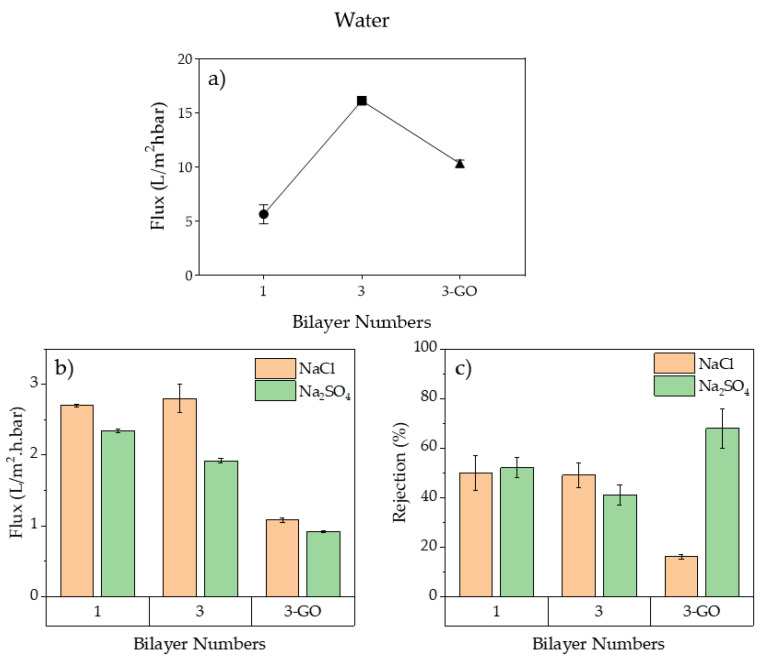
Performance of membranes in crossflow system at 100 psi of working pressure using different feed water solutions: (**a**) water flux of modified membranes with pure water; (**b**) water flux of modified membranes with saline solution; (**c**) rejections of modified membranes with saline solutions.

The membranes’ performances were evaluated through a crossflow filtration test using pure water and two different saline solutions, NaCl and Na_2_SO_4_ 10 mM, as feed solution at a working pressure of 100 psi. First, the water flux of the pure water was determined for the modified membranes with one and three bilayers, (PAA-PEI)/PVDF and (PAA-PEI)_3_/PVDF, respectively. It can be seen that a higher flux was achieved with the membrane modified with three bilayers (Figure 4a). The incorporation of GO produced a decrease in water flux with respect to the membrane without GO when the feed solution was pure water. However, the flux of the (GO/PAA-PEI)_3_/PVDF membrane was higher than that observed for the membrane modified with one bilayer (PAA-PEI)/PVDF (Figure 4a). On the other hand, the (PAA-PEI)/PVDF and (PAA-PEI)_3_/PVDF membranes showed similar water fluxes in filtration tests with NaCl 10 mM and Na_2_SO_4_ 10 mM, but these fluxes were lower than the fluxes observed during pure water filtration (Figure 4b). This behavior could be explained by an electrical double layer produced on the membrane surface due to the presence of ions in the feed solution [68]. Moreover, the membrane modified with GO (GO/PAA-PEI)_3_/PVDF showed a decrease in the water flux in comparison with the membrane modified without GO. This behavior can be explained by the membrane pores being blocked by the GO sheets. In fact, the SEM images of the (GO/PAA-PEI)/PVDF membrane showed a decrease in the porosity on the surface (Figure 2). The membrane obtained in the present study, (PAA-PEI)_3_/PVDF, showed a water flux similar to that reported by Liu et al. for (PAA-PEI)_1.5_/PAN membranes modified with 1.5 bilayers and static deposition and rinsing as the modification method [24], which is a similar method to the one applied in this study. However, our membrane was prepared with a PE concentration and a coating time three times lower than the PE concentration and coating time used by Lui et al. Moreover, the water flux of the membranes in this study was similar to that reported for NF–antibacterial polyamide membranes modified with copper [57,69].

Regarding salt rejections, the supporting UF-PVDF membrane showed a very low rejection of ions, of (5.3 ± 0.5)% and (6.2 ± 0.2) for NaCl and Na_2_SO_4_, respectively. This behavior was expected of the UF support considering that the MWCO of UF-PVDF support is 50 KDa. The filtration performances to different modified membranes are shown in Figure 4c. The NaCl rejection was similar between membranes modified with one and three bilayers, (50 ± 7)% and (49 ± 5)%, respectively. The Na_2_SO_4_ rejection of the (PAA-PEI)_3_/PVDF membrane was slightly lower than that of the (PAA-PEI)/PVDF membrane. The incorporation of GO into the membrane modified with three bilayers (GO/PAA-PEI)_3_/PVDF produced an increase in the Na_2_SO_4_ rejection to up to (68 ± 8)% and decreased the rejection of NaCl down to (16 ± 1)%. These results suggest that GO sheets promote the formation of preferential channels to pass off small ions like Cl^−^ and reject large divalent ions like SO_4_^−2^. The salt rejection rates observed for the membranes obtained in this study suggest that these membranes could be applied to a forward-osmosis filtration system. Kallen et al. reported on the effects of PAN membranes modified by the LBL assembly of PAA and PDDA (poly (diallyldimethylammonium chloride)) with and without GO on the filtration of different mono and divalent salts by a forward-osmosis system [70]. The membranes modified with GO showed a good performance in the system with a raw solution consisting of 1 M Na_2_SO_4_ and deionized water as the feed.

Antibiofilm experiments were carried out on membranes modified with three bilayers with and without GO. The results are shown in the following section.

### 3.2. Antibiofilm and Antibacterial Activity

#### 3.2.1. Antibiofilm Activity

Biofilm assays were performed in order to assess the antibiofilm effect of PDVF derivatives membranes. *Staphylococcus aureus* and *Escherichia coli* were used as bacteria models, considering that *Staphylococcus aureus* is a food-borne pathogen able to form biofilms, it is also one of the most common pathogens in biofilm infections [71]. *S. aureus* has been detected across various environments, indicating its significant adaptive capacity and that the development of a biofilm acts as a protective strategy, thus facilitating their persistence and growth in challenging environmental conditions [72]. *E. coli* has been a fundamental model organism in molecular biology, genetic engineering and industrial microbiology research. *E. coli* biofilms have been identified as a leading cause of various intestinal infections and many industrial processes associated with food processing [73]. The formation of *E. coli* and *S. aureus* biofilms has a significant impact on industrial processes, with negative consequences and subsequent economic losses.

**Figure 5:**
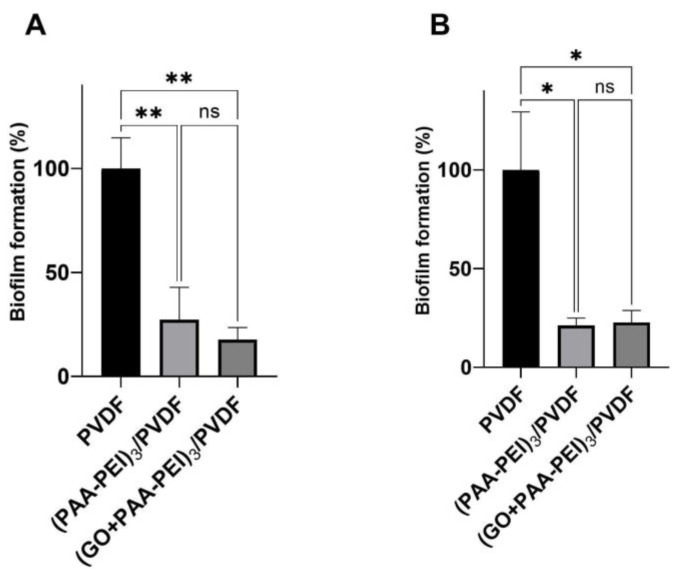
Antibiofilm effects of PVDF derivative membranes. Sterile PVDF membrane pieces were immersed in a culture medium inoculated with *S. aureus*, *E. coli* or an uninoculated medium (control) and incubated for 48 h at 37 °C. Biofilm formation was measured by absorbance at 595 nm and relativized to the control PVDF membrane. Bars represent the mean ± SEM of at least three independent experiments on (**A**) *S. aureus* and (**B**) *E. coli*. Statistical differences were determined using one-way ANOVA followed by Tukey’s multiple comparisons test: * *p* < 0.05, ** *p* < 0.01, ns: not significant.

A significant reduction in biofilm formation carried out by *S. aureus* and *E. coli* was observed in the (PAA-PEI)_3_/PVDF and (GO/PAA-PEI)_3_/PVDF modifications compared to the control (PVDF). No significant differences were found between the two modifications. The (GO/PAA-PEI)_3_/PVDF membrane showed the lowest *S. aureus* biofilm quantification with a reduction of 82.37% compared with control (PDFV membrane, Figure 5A). For *E. coli*, both derivative membranes showed similar biofilm reduction rates with 78.80% for (PAA-PEI)_3_/PVDF and 77.30% for (GO/PAA-PEI)_3_/PVDF (Figure 5B).

A relevant factor contributing to the observed reduction in biofilm formation is the surface roughness of the membranes [54,67]. The membranes modified with three bilayers of PEI and GO ((GO/PAA-PEI)_3_/PVDF) exhibited a significantly lower surface roughness ((27 ± 7) nm) compared to the unmodified membranes ((55 ± 2) nm). This change results in a smoother surface, which limits the initial bacterial adhesion by reducing the number of anchoring points available. The correlation between a reduced roughness and decreased biofilm formation supports the hypothesis that surface topography plays a key role in the antibiofouling capacity of membranes. The SEM images do show a bacterial presence on the modified membranes, but the CFU/cm² data and the reduction in EPS production confirm that many of these bacteria are non-viable, reinforcing the positive effect of a smoother surface in preventing biofouling.

**Figure 6:**
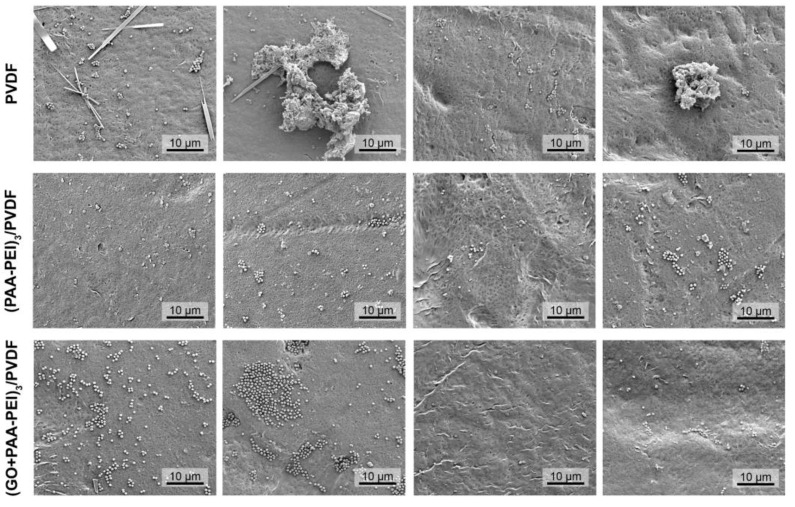
Representative SEM images of the surfaces of the membranes obtained from the in vitro assay for the detection of *Staphylococcus aureus* biofilm. Modified and unmodified membranes were immersed in culture medium inoculated with *S. aureus* and incubated for 48 h at 37 °C, the bacteria were fixed on the surface with glutaraldehyde for visualization. PVDF corresponds to unmodified membranes, while (PAA-PEI)_3_/PVDF and (GO/PAA-PEI)_3_/PVDF correspond to the modified membranes after exposure to the inoculum. All images were taken with a magnification power of 6000×.

**Figure 7:**
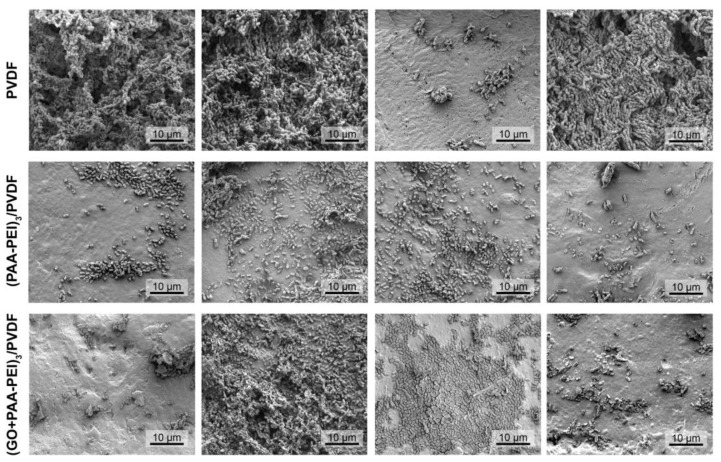
Representative SEM images of the surface of the membranes obtained from the in vitro assay for the detection of *Escherichia coli* biofilm. Modified and unmodified membranes were immersed in culture medium inoculated with *E. coli* and incubated for 48 h at 37 °C, the bacteria were fixed on the surface with glutaraldehyde for visualization. PVDF corresponds to unmodified membranes, while (PAA-PEI)_3_/PVDF and (GO/PAA-PEI)_3_/PVDF correspond to the modified membranes after exposure to the inoculum. All images were taken with a magnification power of 6000×.

In addition, the SEM images provide detailed insights into the morphology and topography of membranes, as well as the formation of biofilms [74]. The SEM analysis showed that *S. aureus* formed a sparse biofilm on the PVDF membrane, characterized by superficial colonization and the development of aggregates with a higher content of what appeared to be exopolysaccharides (Figure 6). An analysis of the biofilm on the modified membranes, both (PAA-PEI)_3_/PVDF and (GO/PAA-PEI)_3_/PVDF, showed that bacteria settled on the membrane surfaces. While the number of bacteria did not appear reduced compared to the control, a change in the aggregation pattern was evident. On the modified membranes, bacterial aggregates decreased, resulting in a form of colonization where microorganisms directly colonized the surface. A similar effect was observed with the biofilm formed by *E. coli* (Figure 7). The biofilm on the unmodified PVDF was thick (Supplementary Figure S2), complex and largely embedded in the EPSs across most of the exposed surface. In contrast, the modified membranes showed only a few isolated clusters of bacteria, which were much smaller in magnitude compared to the biofilm on the unmodified membrane (Supplementary Figure S2). The colonization on these modified surfaces appeared to be more heterogeneous, with microorganisms distributed without being fully embedded in EPSs. SEM images suggest that bacterial adhesion between the unmodified and modified PVDF membranes was similar and the main difference was in the EPS architecture. Guanyu et al. (2020), showed that PEI-coated ion-exchange membranes exhibited no significant reduction in cell attachment but demonstrated a 66% decrease in biofilm formation [75]. This suggests a similar trend in both studies, where surface modifications, such as PEI coatings, primarily impact biofilm formation rather than the initial bacterial attachment.

**Figure 8:**
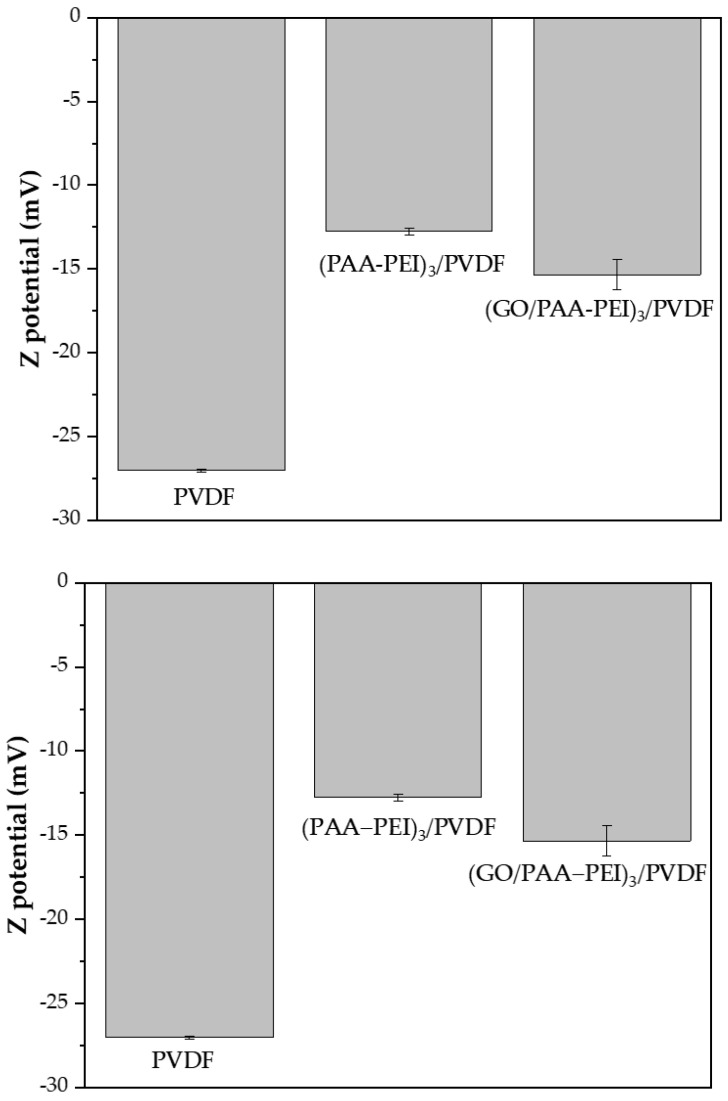
Z potentials of membranes at pH 7.

Among the different strategies reported to control the formation of biofilms on membrane surface, we highlight increases in hydrophilicity and changes in the membrane’s surface charges. The first strategy aims to avoid bacterial adhesion due to poor interactions with hydrophobic cell membranes. The second promotes electrostatic repulsion between the membrane surface and the bacteria that have a cell membrane with a charge similar to the membrane surface. In order to determine the surface physicochemical properties of the membranes that influences the antibiofilm effect, the Z potentials of the unmodified and modified membranes were determined. The Z potential of membranes at pH 7 are shown in Figure 8, in which it is possible to observe that the PVDF control membrane showed a more negative Z potential (−27.04 ± 0.07 mV) whilst the modified membranes, (PAA-PEI)_3_/PVDF and (GO/PAA-PEI)_3_/PVDF, showed Z potentials of (−12.8 ± 0.2) mV and (−15.4 ± 0.9) mV, respectively.

The PVDF membrane showed the highest biofilm formation despite this membrane showing a more negative surface than the modified membranes (Figure 6 and Figure 7). This behavior could be attributed to the more hydrophobic character of the PVDF membrane’s surface which allows for increased interactions with hydrophobic foulants including microorganisms in comparison with the modified membranes, which showed more hydrophilic surfaces (Figure 1), considering that the antibiofilm properties are produced by a combination of different physicochemical surface effects. On the other hand, (GO/PAA-PEI)_3_/PVDF showed the best anti-biofilm performance against both model bacteria. In this case, both modified membranes showed a similar hydrophilicity; hence, a difference in the biofilm developing on the surface could be due to the surface charge and roughness, considering that (GO/PAA-PEI)_3_/PVDF showed a more negative and smoother surface. Finally, PEI and GO have been reported to brr antimicrobial materials for this reason, as studies on the antibacterial effects of the modified membranes were developed in order to evaluate the cell viability of the bacteria.

#### 3.2.2. Antibacterial Activity

To evaluate the antibacterial activity of PVDF derivative membranes, the membranes were incubated with *S. aureus* or *E. coli* for 24 h. Following incubation, the bacteria were recovered from the membranes, and the percentage reduction was calculated. The (PAA-PEI)_3_/PVDF and (GO/PAA-PEI)_3_/PVDF membranes were able to reduce the number of *S. aureus* by 99.98%. However, these membranes were less efficient in reducing *E. coli*, with reduction percentages ranging between 67.42% and 93.05% (Table 1).

Membrane composition can help to reduce the microbial load through antimicrobial activity. Graphene and GO exhibit significant antimicrobial properties, which have led to their widespread use in materials requiring antibiofouling characteristics. The antibacterial effects of graphene are primarily attributed to its sharp edges, which mechanically disrupt bacterial membranes, leading to cell lysis and the leakage of intracellular cytoplasm [41]. Additionally, GO generates oxidative stress through the production of reactive oxygen species (ROS), including hydroxyl radicals and superoxide anions. These reactive molecules disrupt essential cellular structures such as lipids, proteins, and nucleic acids, leading to irreversible damage in bacterial cells. This oxidative damage, coupled with the distinct structural attributes of GO, enhances its effectiveness as an antibacterial agent against both Gram-negative and Gram-positive bacteria [41,42]. The antimicrobial activity of GO has been explored by various research groups. For instance, Zhang et al. [76] demonstrated that the modification of cellulose acetate membrane filters with GO showed inhibitory effects on *S. aureus* and *E. coli,* with a 51.8% and 31.6% reduction, respectively [76]. In addition, Kaneda et al. (2019) showed PVDF and polysulfone membranes modified with GO via photo-grafting, achieving a strong antibacterial activity against *E. coli*, reducing viable cell counts by 90% and 75%, respectively [77]. These findings support our observations, suggesting that modifying filtration membranes with GO enhances their antimicrobial effects.

On the other hand, PEI has been shown to exhibit significant cytotoxicity, primarily through the induction of apoptosis and necrosis, effects that have been determined in eukaryotic cell lines. Its strong cationic charge enables it to bind to DNA, forming PEI/DNA complexes that can lead to DNA damage and the disruption of cellular membranes [78,79]. Furthermore, PEI has demonstrated a selective antimicrobial activity, with one study reporting a greater efficacy against *S. aureus* compared to *E. coli*, likely due to structural differences in their bacterial membranes [80]. In addition to these antimicrobial mechanisms, PEI exhibits antifouling properties by enhancing steric hindrance and forming hydration layers on the membrane surface. These layers act as physical barriers, reducing the adsorption of proteins and other organic fouling agents [81]. The antifouling performance is further enhanced when PEI is combined with PAA. PAA introduces carboxylic groups that contribute to hydration layers, creating a dual mechanism where steric hindrance from PEI and hydration barriers from PAA work together to minimize protein adsorption and bacterial attachment. This combined effect improves the resistance of the membrane to both organic and microbial fouling.

Once the cells were irreversibly attached to the surface, they began producing EPSs, forming a biofilm structure that could completely block the membrane’s pores [15]. Although the membrane modifications suggested do not affect the number of cells on the surface of the membranes (Figure 6 and Figure 7), the reduced biofilm formation (Figure 5) can be attributed to the pronounced effect of the modified membranes on bacterial viability (Table 1), thereby hindering their ability to form a mature biofilm. The SEM images reveal a substantial bacterial presence in certain sections of the modified membranes; however, viable cell quantification assays show lower bacterial counts compared to the control membranes. This discrepancy suggests that many of the bacteria visible in the SEM images may not be viable and are, therefore, incapable of producing EPSs. While SEM images provide a visualization of bacterial adhesion, the reduction in CFU/cm² counts implies that a significant proportion of these bacteria may be dead or inactive. This highlights the importance of distinguishing between the total bacterial presence observed in SEM images and the viable population capable of developing a biofilm. We attempted to use viability staining to assess the presence of viable bacteria within the biofilm. In *E. coli*, a higher number of non-viable bacilli were detected on the modified membranes compared to the control (Supplementary Figure S2). This observation indicates that the surface modifications may influence bacterial viability, potentially contributing to reduced bacterial survival. The combined use of GO, PAA and PEI in the modified membranes introduces a multifaceted approach to biofilm control. GO disrupts bacterial membranes and generates oxidative stress, while PAA and PEI synergistically minimize bacterial adhesion and EPSs production through steric and hydration barriers. This combination ensures that even when bacterial cells are present, their ability to form mature biofilms is significantly impaired, further enhancing the antibiofilm performance of the membranes. These results are consistent with the CFU counts, suggesting that while bacterial adhesion is not entirely prevented, the membranes significantly reduce bacterial proliferation and biofilm formation.

The formation of biofilms, specifically the aggregation of EPSs, can significantly reduce membrane performance by affecting permeate flux and salt rejection, indicating the need to clean the membranes. Physical cleaning uses mechanical or hydraulic forces to remove the compound, while chemical cleaning employs agents to eliminate irreversible fouling. Chemical cleaning can be performed in situ (with the membrane in place) or ex situ (by rinsing removed membranes). However, chemical cleaning is often ineffective and cannot completely eradicate mature biofilms [82]. For example, biofilms have been shown to tolerate concentrations of antibiotics and disinfectants 100–1000 times higher than those required to eliminate planktonic cells [83]. The changes observed in the biofilm morphology and bacterial viability on our modified membranes could be crucial when applying treatments aimed at eliminating biofouling.

## 4. Conclusions

In this study, we successfully developed modified PVDF membranes with polyethylenimine (PEI), poly(acrylic acid) (PAA) and graphene oxide (GO) through layer-by-layer (LbL) assembly to enhance their antibacterial and antibiofilm properties. The results showed that the addition of one bilayer of PEI and PAA significantly improved the membranes’ hydrophilicity, but a successive bilayer coating up to a total of three bilayers produces a decrease in hydrophilicity, with the (PAA-PEI)_3_/PVDF membrane showing the highest contact angle. Incorporating GO into the PAA-PEI bilayers further enhanced the hydrophilicity of the membrane, while the SEM and AFM analyses revealed a honeycomb-like morphology and smoother surface, which is favorable for reducing bacterial adhesion and biofilm formation.

The modified membranes showed a significant reduction in biofilm formation and bacterial viability against *S. aureus* and *E. coli*, while maintaining good water flux and salt rejection performances. The SEM analysis suggested that although bacterial adhesion was not entirely suppressed, the modified membranes hindered biofilm maturation by reducing bacterial proliferation and the production of extracellular polymeric substance (EPSs). The reduction in CFU counts, despite a visible bacterial presence on the membrane’s surfaces, supports the idea that many of these bacteria were non-viable. These findings suggest that the developed membranes, particularly the (GO/PAA-PEI)_3_/PVDF membrane, are effective for preventing biofouling and enhancing membrane lifespan, thus holding great potential for applications in water treatment and other industrial applications.

## 5. Patents

The membranes modified to exert an antibiofilm activity and their potential uses are being patented in Chile under the patent application number 202403211 (INAPI, Chile)

## Figures and Tables

**Table 1:** Antimicrobial efficacy of PVDF and modified PVDF membranes against *S. aureus* and *E. coli*.

	*Staphylococcus aureus*	*Escherichia coli*
Inoculum	Log cfu/mL	
	5.75	5.29
	PVDF membrane	
Contact time	Mean log cfu/cm^2^	
0 h	5.17	4.8
24 h	5.02	4.9
	(PAA-PEI)_3_/PVDF membrane	
	Mean log cfu/cm^2^	
Contact time 24 h	1.29	3.75
Microbial kill (% reduction)	99.98%	93.05
	(GO + PAA-PEI)_3_/PVDF membrane	
	Mean log cfu/cm^2^	
Contact time 24 h	1.32	4.42
Microbial kill (% reduction)	99.98%	67.42%

## Data Availability

The original contributions presented in the study are included in the article and Supplementary Material, further inquiries can be directed to the corresponding author.

## Supplementary Materials

The following supporting information can be downloaded at: https://www.mdpi.com/article/10.3390/polym16233418/s1, Figure S1: ATIR spectra of the PVDF membrane and PVDF membranes modified. Light green zone higlight peaks correspond to PVDF; Figure S2: Representative SEM images of an overview of the membrane surface after in vitro incubation with *Escherichia coli*. These SEM images were selected to show the volume of the biofilm formed over the surface of the modified and unmodified membranes after immersed in culture medium inoculated with *E. coli* and incubated for 48 hours at 37°C. PVDF corresponds to unmodified membranes, while (PAA−PEI)_3_/PVDF and (GO+PAA −PEI)_3_/PVDF correspond to the modified membranes after exposure to the inoculum; Figure S3: Determination of live and dead bacteria within biofilm. A. Confocal microscopy of *E. coli* biofilms on membranes evaluated using the LIVE/DEAD assay. PVDF corresponds to unmodified membranes, while (PAA−PEI)_3_/PVDF and (GO+PAA −PEI)_3_/PVDF correspond to the modified membranes after exposure to the inoculum. Green colour indicate live cells, and red colour indicate dead cells. B The quantification of live cells is presented as a percentage of the total cells in each field. The data represent the average of 5 independent fields of view. Statistical analysis was performed using one-way ANOVA, followed by Tukey’s multiple comparisons test. Statistically significant differences were observed between the tested conditions. **** indicates a *p* value < 0.0001; Figure S4: Zones of the droplet for contact angle measurements.
