# Supplementary material for: Antibiofilm Effects of Modifying Polyvinylidene Fluoride Membranes with Polyethylenimine, Poly(acrylic acid) and Graphene Oxide
Source: Polymers (Basel). 2024 Dec 5;16(23):3418. doi: 10.3390/polym16233418 (PMC11644558; doi:10.3390/polym16233418)
Supplement: Supplementary file 1 [file polymers-16-03418-s001.zip › polymers-3315134-supplementary.pdf]

polymers

MDPI

Supementary Material

Antibiofilm Effects of Modifying Polyvinylidene Fluoride Membranes with Polyethylenimine, Poly(acrylic acid) and Graphene Oxide

Mario Castillo-Ruiz [1,2], Constanza Negrete [3], Juan Pablo Espinoza [4], Iván Martínez [5], Leslie K. Daille [6], Christopher González [7] and Bárbara Rodríguez [7,*]

[1] Escuela de Tecnología Médica, Facultad de Ciencias de la Salud, Universidad Bernardo O'Higgins, General Gana 1702, Santiago 8370854, Chile; mario.castillo@ubo.cl
[2] Escuela de Química y Farmacia, Facultad de Medicina, Universidad Andres Bello, Sazié 2320, Santiago 8370134, Chile
[3] Facultad de Ciencias Naturales, Matemáticas y del Medioambiente, Universidad Tecnológica Metropolitana, Las Palmeras 3360, Ñuñoa 7800003, Chile; constanza.negretea@utem.cl
[4] CIBQA, Facultad de Ciencias de la Salud, Universidad Bernardo O'Higgins, Fábrica 1865, Santiago 8320000, Chile; jespinoza@ubo.cl
[5] Departamento de Ciencias Químicas y Biológicas, Facultad de Ciencias de la Salud, Universidad Bernardo O'Higgins, General Gana 1702, Santiago 8370854, Chile; ivan.martinez@ubo.cl
[6] Centro GEMA-Genómica, Ecología & Medio Ambiente, Universidad Mayor, Camino La Pirámide 5750, Santiago 8580745, Chile; lkdaille@gmail.com
[7] CIRENYS, Escuela de Química y Farmacia, Facultad de Ciencias Médicas, Universidad Bernardo O'Higgins, General Gana 1702, Santiago 8370854, Chile; gonzalezponcechristopher@gmail.com
* Correspondence: barbara.rodriguez@ubo.cl

S1. ATR-FTIR of Membrane Surface

The chemical composition of the PVDF membrane and its subsequent modifications was characterized by ATR-FTIR, and the spectra are shown in Figure 1. The PVDF membrane showed the typical peaks for the PVDF polymer at 1400 cm^{-1} and 1170 cm^{-1} for the C-H stretching vibration, the amorphous phase at 836 and 875 cm-1. Bonds at 836, 1170 and 1275 are all representative of the C–F bonds present in PVDF [*Membranes* **2018**, *8*(3), 70].In addition, the PVDF phase was identified, where exclusive peaks for the β phase were found at 445, 470 ,1275, 1431 cm^{-1} [RSC Adv., 2017, 7, 15382]. These peaks are also present in the modified membranes.

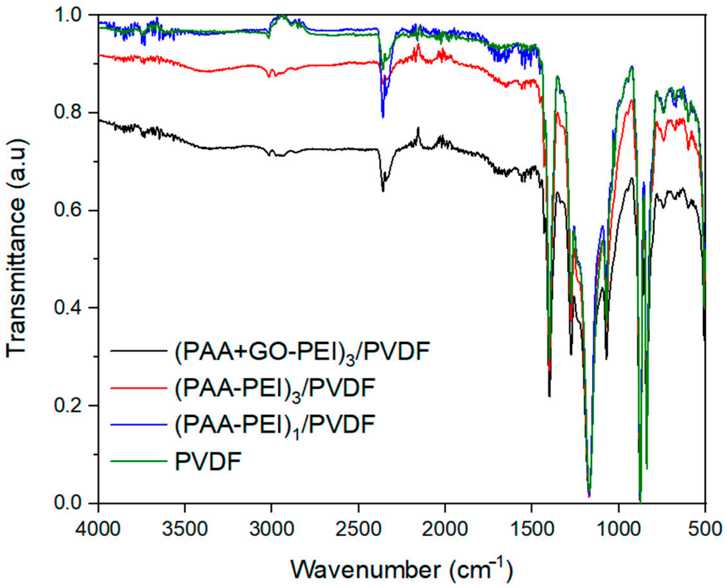

Figure S1. ATIR spectra of the PVDF membrane and PVDF membranes modified. Light green zone higlight peaks correspond to PVDF.

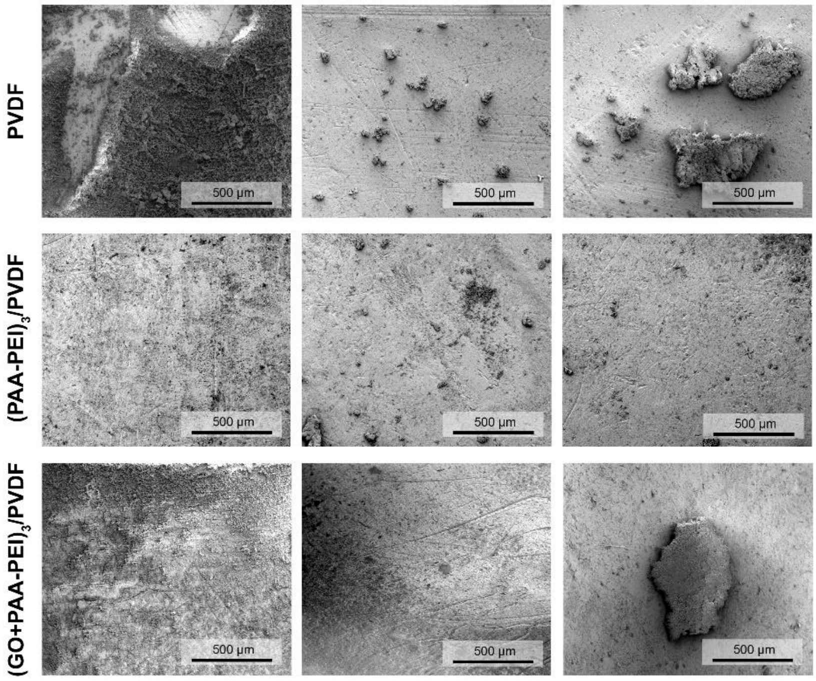

Figure S2. Representative SEM images of an overview of the membrane surface after *in vitro* incubation with *Escherichia coli*. These SEM images were selected to show the volume of the biofilm formed over the surface of the modified and unmodified membranes after immersed in culture medium inoculated with *E. coli* and incubated for 48 hours at 37°C. PVDF corresponds to unmodified membranes, while (PAA-PEI)3/PVDF and (PAA+GO-PEI)3/PVDF correspond to the modified membranes after exposure to the inoculum.

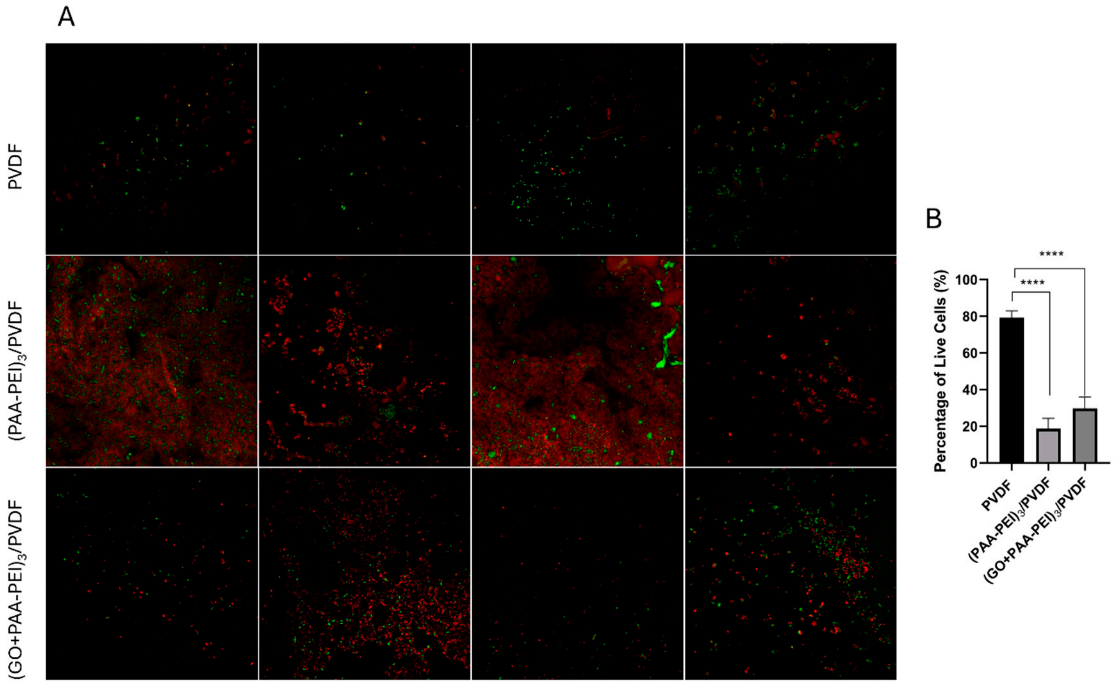

Figure S3. Confocal microscopy of *E. coli* biofilms on membranes evaluated using the LIVE/DEAD assay. PVDF corresponds to unmodified membranes, while (PAA-PEI)3/PVDF and (PAA+GO-PEI)3/PVDF correspond to the modified membranes after exposure to the inoculum. Green colour indicate live cells, and red colour indicate dead cells. B The quantification of live cells is presented as a percentage of the total cells in each field. The data represent the average of 5 independent fields of view. Statistical analysis was performed using one-way ANOVA, followed by Tukey's multiple comparisons test. Statistically significant differences were observed between the tested conditions. **** indicates a p value < 0.0001.

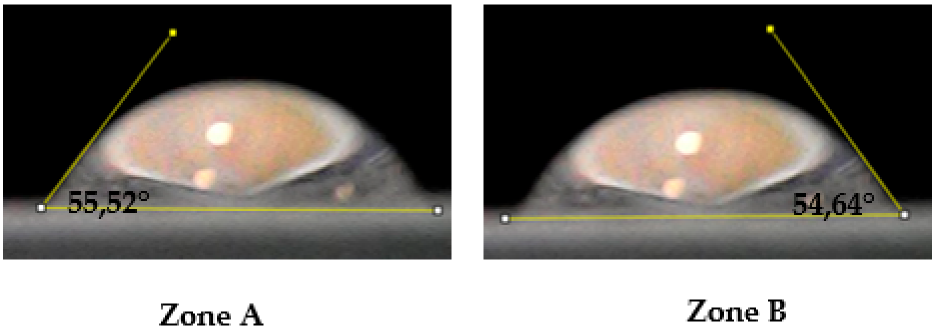

Figure S4. Zones of the droplet for contact angle measurements.
